# Could artificial intelligence gradually replace classical adjuvants?

**DOI:** 10.3389/fimmu.2026.1710109

**Published:** 2026-01-28

**Authors:** Jose G. Marchan-Alvarez

**Affiliations:** 1 Department of Women’s and Children’s Health, Karolinska Institutet, Stockholm, Sweden; 2 Astrid Lindgren Children’s Hospital, Stockholm, Sweden

**Keywords:** adjuvants, AI, artificial intelligence, immunogenicity, vaccine design, replacement, substitution

## Abstract

Adjuvants have been indispensable in vaccinology, enhancing immunogenicity, shaping adaptive immune polarization, and extending the durability of protective responses. Classical adjuvants, including alum, oil-in-water emulsions, liposomal formulations, and toll-like receptor agonists, function by fueling innate immunity, promoting antigen presentation, and modulating cytokine milieus. Yet, these compounds face persistent limitations such as reactogenicity, species-specific responses, manufacturing complexity, and regulatory barriers. Artificial intelligence (AI) and core subfields such as machine learning are revolutionizing vaccine design by enhancing antigen engineering, delivery system optimization, immunogenicity modeling, and *in silico* screening of novel immune potentiators. Here, I propose a speculative yet testable hypothesis: AI-driven design of potent antigens may progressively replace classical adjuvants. By embedding adjuvant-like functions directly into computationally designed antigens, AI-designed vaccines could replicate or excel the mechanistic roles of traditional adjuvants. This perspective critically explores current AI applications in vaccinology, discusses key challenges in the field, including biological complexity, data limitations, safety, and regulatory and ethical considerations, and outline the spectrum of adjuvant replacement—from augmentation to full substitution. Finally, a roadmap is proposed for transitioning toward AI-driven adjuvant surrogates. Notably, this work highlights a potential paradigm shift in vaccine design, suggesting that future advances will lead to a gradual, stepwise transition from the use of traditional adjuvants to computationally guided antigens and delivery platforms capable of outperforming classical adjuvant functions, thus creating a new vaccine era without exogenous chemical additives.

## 1 Introduction

Adjuvants remain fundamental components of modern vaccinology (1). Since the introduction of insoluble aluminum salts (hereafter known as alum) in the 1920s, adjuvants have enhanced immunogenicity, shaped T helper polarization, and extended the durability of protective immunity (1–5). However, classical adjuvants remain imperfect. For instance, they are often associated with reactogenicity, species-specific differences, and regulatory barriers that slow approval of new formulations (6).

**Figure 1:**
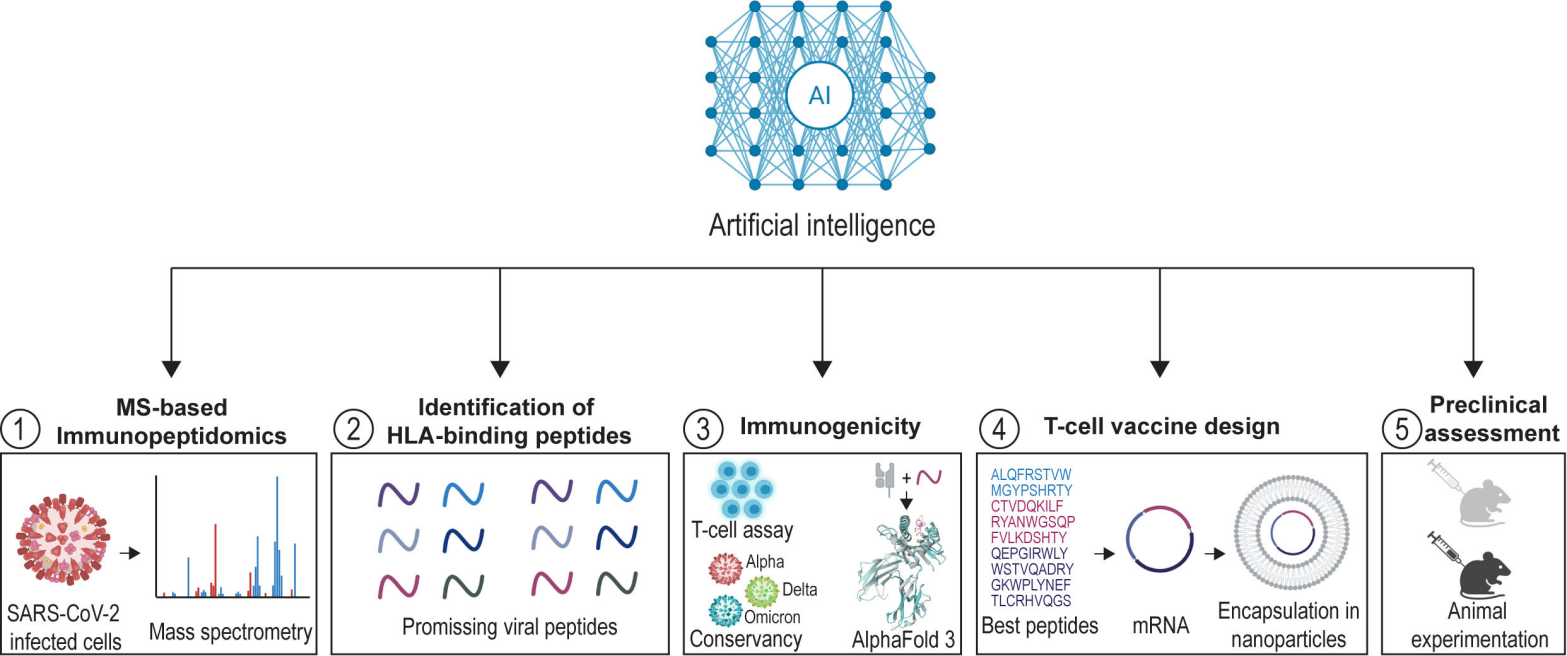
Integration of artificial intelligence (AI) within immunopeptidomics pipelines to guide next-generation T cell vaccine design against SARS-CoV-2. Viral peptides naturally presented by HLA molecules are identified using high-resolution mass spectrometry (MS), followed by computational analysis leveraging artificial intelligence and deep learning algorithms to match spectra against reference databases and identify or predict candidate T-cell peptides. The immunogenic potential of these peptides is then assessed through experimental assays. The recent and groundbreaking development of AlphaFold 3 can support structural assessment of peptide-HLA biomolecular complexes. Subsequently, top-performing peptides are incorporated into mRNA vaccine platforms and formulated with AI-improved nanoparticle-based delivery systems for *in vivo* administration in preclinical models. Figure made with Biorender.com and Illustrator.

The rapid development of artificial intelligence (AI) and relevant subfields such as machine learning represent novel avenues for vaccine design. Initially deployed to accelerate epitope prediction and antigen selection, AI now branches through nearly every stage of the vaccine pipeline (**Figure 1**): from predicting immunogenicity and modeling immune system dynamics, to optimizing formulations and identifying novel small-molecule immunostimulants (7, 8). Far from being niche tools for computational specialists, AI-based platforms are now becoming transformative engines that could redefine the very principles of vaccinology (7–9).

Here, I raise a speculative yet testable hypothesis: AI-guided antigen design may progressively replace the bioactive properties of classical adjuvants. “Replacement” does not imply that AI functions as a chemical additive. Rather, it refers to the possibility that computationally designed antigens, delivery systems, and immune programming strategies can surpass the need for traditional adjuvants by creating vaccines with remarkable immunogenicity. In particular, the direct incorporation of adjuvant-like motifs into the vaccine constructs (8), together with improvements in nanoparticle-based delivery vehicles (10), could enhance the robustness, breadth, and durability of the immune response. Thus, the concept of future AI-powered vaccines challenges the conventional view that exogenous adjuvants are permanent companions of vaccine formulations. Please note that the goal of this hypothesis is not to diminish the vital role of adjuvants in tackling infectious threats (11), but to envision an upcoming future in which AI makes them dispensable in a gradual manner.

Accordingly, this article addresses the fundamental roles of adjuvants in vaccinology, the current state of AI in the field, and the potential for AI-designed antigens to progressively replace typical adjuvants. Moreover, it outlines the challenges and strategies required to realize this vision. Ultimately, this article aims to encourage both computational scientists and immunologists to move beyond incremental optimization and embrace an eventual paradigm shift in vaccine design: one where the boundaries between adjuvant and algorithm begin to blur.

## 2 The fundamental roles of adjuvants in modulating immunity.

The word adjuvant derives from the Latin *adjuvare*, meaning “to help” and indeed adjuvants have historically been viewed as the “helpers” of vaccinology—substances that potentiate immune responses to co-administered antigens (11, 12). Since the discovery of alum’s immunopotentiating properties nearly a century ago, a limited repertoire of adjuvants has been incorporated into licensed vaccines, including oil-in-water emulsions (MF59, AS03), liposomal formulations (AS01), and defined pathogen-associated molecular patterns (PAMPs) such as toll-like receptor (TLR) agonists (e.g., CpG 1018) (1, 11, 13). However, these diverse compounds share several mechanisms that are yet to be fully understood. Below, I provide an overview of the current insights into these principles. For an expanded coverage of adjuvant biology and applications, please see references (1, 11).

### 2.1 Recognition of highly conserved targets and activation of innate immunity.

A primary role of adjuvants is to activate the innate immunity through pattern recognition receptors (PRRs) including TLRs, retinoic acid-inducible gene I (RIG-I), the stimulator of interferon genes (STING) protein, C-type lectins, nucleotide-binding oligomerization domain (NOD)-like receptors (NLRs), and inflammasome receptors such as NLRP3. These innate sensors recognize conserved PAMPs in microbes as well as critical regions in adjuvants, thus leading to the production of type I interferons, proinflammatory cytokines, and chemokines that recruit and activate immune effector cells (11).

### 2.2 Antigen presentation and activation of adaptive immunity

Adjuvants frequently increase microbial protein delivery to antigen-presenting cells, including dendritic cells, B cells and macrophages. For example, the emulsion-based adjuvant MF59 promotes the recruitment of dendritic cells and enhances antigen internalization, thereby increasing major histocompatibility complex (MHC, also known as human leukocyte antigen system or HLA) class I and II presentation, which directs the priming of both CD8^+^ and CD4^+^T-cell responses, respectively (14–16).

### 2.3 T-cell polarization

Different adjuvants imprint distinct cytokine milieus that bias adaptive responses toward CD4^+^ T-cells such as T helper (TH) 1 and 2 cells or T follicular helper phenotypes. Alum, for instance, is associated with Th2-skewing, whereas AS01 drives robust Th1 and cytotoxic T-cell responses. Such polarization is crucial in tailoring immunity against intracellular pathogens, extracellular microbes, or tumor antigens (11).

### 2.4 Depot and delivery effects

Many adjuvants also serve as delivery vehicles that retain antigen at the injection site or release it in a sustained manner. For example, alum forms antigen depots that prolong exposure and liposomal compounds can co-deliver antigens directly to antigen presenting cells, as mentioned above. These physicochemical properties are increasingly exploited in rational adjuvant design (1, 11).

While indispensable in most of vaccine formulations to date, classical adjuvants have several limitations (6), including: i) reactogenicity or adverse events such as pain, redness, swelling, fever, myalgia, headache, rash, and in extreme cases anaphylactic reactions and autoimmune diseases; ii) species-specific responses that complicate preclinical evaluation and limit predictive validity of animal models; iii) manufacturing scale-up, formulation stability, and cold-chain requirements; and iv) rigorous regulatory approval, with only a handful licensed globally in the past three decades.

In summary, adjuvants provide a variety of critical functions, from innate activation to antigen delivery, that are central to vaccine design (11). However, their drawbacks highlight opportunities to explore other strategies. It is within this area that AI may eventually step in, not merely to optimize adjuvant selection, but to replicate or fully replace many of these mechanistic principles by computationally designed antigens and delivery systems.

## 3 AI-assisted design and optimization of vaccines and adjuvants.

### 3.1 Computationally enhanced immunopeptidomics

Immunopeptidomic approaches, particularly mass spectrometry, are reshaping the discovery of MHC-associated peptides, promoting direct identification of conventional and rare peptides with robust immunogenicity that have been used in vaccine design (17). In line with this, current machine learning and deep learning models are increasingly used to predict peptide fragmentation spectra from amino acid sequences, which in turn improve sensitivity and specificity in proteomic analyses and facilitate immunopeptidome discovery from large search spaces such as those created by post-translational modifications or non-tryptic peptides. Furthermore, deep learning frameworks are being trained to assess spectrum quality, cluster spectral features, and automate spectral interpretation, which reduces false positives and accelerates throughput (18). Notably, recent studies in AI-assisted mass spectrometry-based analytical methods have also led to accurate quantification of adjuvants and their degradation products in complex liposomal formulations, as observed with the saponin adjuvant QS-21 (19). Such high-resolution quantitative data (20) yield a valuable foundation for AI-guided vaccine design, as their integration into predictive models may inform adjuvant stability, support rational optimization of liposomal formulations, guide the selection of adjuvant-antigen combinations and assess reactogenicity and safety of novel vaccine models.

### 3.2 Databases for epitope prediction

Early applications of AI and machine learning models in vaccinology were primarily directed toward improving the prediction accuracy of MHC-associated peptides for B-cell and T-cell activation compared with traditional motif-based approaches (8). Now, deep learning architectures predict conformational epitopes and estimate immunogenic potential (9, 21, 22). Notably, curated repositories trained on large experimental datasets (e.g., IEDB) also incorporate innate immune recognition features, such as predicted binding to TLRs (13, 23), interferon type I production (24), proteasomal processing (25) and TAP (transporter associated with antigen processing) transport efficiency (26), which improve the prediction of MHC-associated peptides for new vaccines that may lead to better immune responses (27, 28).

### 3.3 Protein engineering and three-dimensional modeling

Generative adversarial networks and transformer-based protein design (e.g., AlphaFold 3 and AlphaFold-inspired systems) can enhance the creation of antigens with optimized structural stability, improved expression, and embedded immunostimulatory motifs (29, 30). One emerging strategy involves engineering antigens with PAMP-like elements that are sensed by TLRs, RIG-I, STING, and other PRRs, merging adjuvant and antigen functions in a unified construct. Moreover, the very recent discovery that proteasome-derived peptides contribute to cell-intrinsic antimicrobial immunity (31) offers new opportunities for rational vaccine design. For example, by combining advances in proteasome cleavage prediction and immunopeptidomics with AI-driven modeling, antigen sequences can be optimized to selectively produce both high-affinity MHC-binding epitopes and immunostimulatory defense peptides, enhancing immunogenicity while limiting unintended effects. Importantly, as the MHC presentation of antigenic peptides is necessary to elicit immune responses, the peptide-MHC biomolecular complex formation also represents a critical consideration in vaccinology. In this context, the outstanding development of AlphaFold 3 (30) can support a rapid and accurate assessment of peptide-MHC complex formation together with associated T-cell receptors, thus supporting the immunogenicity potential of vaccine candidates, an advantage that is particularly critical during public health emergencies (32, 33).

### 3.4 *In silico* design of delivery vehicles with adjuvant-like properties

Beyond antigen design, AI helps in predicting the performance of nanoparticle carriers (20) and liposome formulations (10, 34). Machine learning models thus integrate physicochemical features (size, charge, composition) with biological data (biodistribution, cellular uptake) to identify delivery vehicles that enhance antigen presentation and control immune activation (35). These approaches reduce the empirical screening of formulations and moves toward rationally designed delivery systems with built-in adjuvant functionality.

### 3.5 Data-driven prediction of immunogenicity

In recent years, machine learning models trained on systems vaccinology datasets have been developed to predict antibody titers, cytokine profiles, T-cell polarization, and durability of immune responses (9, 36). By modeling downstream immune outcomes, these tools can help to anticipate the qualitative “adjuvant-like effects” that classical formulations typically provide. Additionally, some frameworks predict adverse events, reactogenicity, and toxicity (37), offering an avenue to replace trial-and-error adjuvant testing with *in silico* safety assessment.

### 3.6 AI-based cells

From a more radical perspective, “virtual” or “digital” cell models (38, 39) are a promising framework for evaluating AI-designed antigens that aim to incorporate adjuvant-like functions. These *in silico* systems, which initially relied on differential equation-based and stochastic approaches to simulate defined cellular contexts (39), can in theory be extended to explore the spatiotemporal dynamics of innate immune sensing, antigen processing, and downstream immune activation. By enabling systematic interrogation of antigen-intrinsic immunostimulatory properties, virtual cell tools could complement experimental studies and reduce the time and resources required for early-stage evaluation (38, 39). However, a comprehensive AI virtual cell configuration specifically tailored to murine or human immunology has yet to be developed, and the reliable application of such models will require, of course, substantial methodological improvements and rigorous validations before they can lead to predictive insights relevant to adjuvant replacement and vaccinology.

These exciting advances, together with emerging AI powerful technologies, illustrate how AI can help to speed up the discovery of novel adjuvant candidates and embed them directly into better vaccine candidates, suggesting that the role of classical adjuvants may rapidly decrease as computationally designed antigens and delivery systems reach their prime.

## 4 Shaping the concept of replacement of adjuvants with AI-powered vaccines

The bold speculation that artificial intelligence could “replace” adjuvants requires careful consideration. Adjuvants are not single-purpose additives; rather, they have a constellation of functions, including activation of innate immune sensors, modulation of adaptive immune responses, enhancement of antigen presentation, and optimization of antigen delivery (11), as mentioned earlier. For future AI-powered vaccines to eventually assume these roles, they must achieve outcomes that are equivalent or superior across these immune functions while maintaining safety profiles. Therefore, replacement should be viewed not as a binary or abrupt transition, but as a progressive, overlapping spectrum of functionalities. Within this framework, several potential areas of replacement can emerge: mimicry, reduction, augmentation, and full replacement.

### 4.1 Mimicry

Mimicry represents a central strategy in which AI-designed vaccine constructs internalize classical adjuvant functions. Experimental evidence supports the feasibility of this approach across multiple pathogens, including influenza, severe acute respiratory syndrome coronavirus 2 (SARS-CoV-2), and monkeypox. mRNA vaccine platforms exemplify this concept, as they intrinsically engage innate immune sensors such as TLR7/8 and RIG-I/MDA-5 through sequence- and structure-dependent RNA motifs, contributing to immunogenicity even in the absence of conventional adjuvants (40). Similarly, synthetic CpG-containing sequences embedded within antigen constructs have been shown to activate TLR9 signaling (41), leading to antigen-specific B-cell and T-cell responses in preclinical models. Beyond nucleic acid-based vaccines, protein and nanoparticle platforms also illustrate adjuvant mimicry (42, 43). Virus-like particles and self-assembling nanoparticles, such as ferritin-based scaffolds, can promote innate immune activation through repetitive antigen display and efficient uptake by antigen-presenting cells, resulting in enhanced germinal center responses and durable antibody production (43). In parallel, conjugation or co-encoding of STING-activating motifs, such as 2′3′-cyclic GMP-AMP (2′3′-cGAMP), within antigen constructs has been shown experimentally to potentiate CD8^+^ T-cell responses by coupling antigen presentation with localized innate signaling (44).

### 4.2 Reduction

In parallel, the above insights can also offer a pathway to reduce, rather than eliminate, dependence on potent external adjuvants. Engineered nanoparticle platforms, for example, can markedly improve antigen uptake, cross-presentation, and CD8^+^ T-cell activation, thereby achieving dose-sparing effects (45). These polymeric nanoparticles could substantially improve therapeutic efficacy against tumors, indicating that optimized delivery vehicles alone can reduce the need for traditional adjuvants (45). Similarly, self-assembling peptide-based nanovaccines promote efficient dendritic cell activation and lymph node targeting elicit potent antigen-specific cytotoxic responses without supplemental adjuvants (10).

### 4.3 Augmentation

The overlapping examples in 4.1 and 4.2 show that at the most basic level of functional enhancement AI and machine learning are already being used to augment traditional vaccine adjuvants by optimizing how they are paired with antigens and how dosing regimens are structured to balance immunogenicity and safety. Rather than replacing adjuvants outright, these computational approaches can improve the synergy between antigen and adjuvant, identify optimal combinations with superior immune profiles, and refine schedules to elicit robust and durable responses (46). Machine learning has been applied in human vaccine studies to distinguish immune signatures associated with different adjuvant formulations and to identify correlates of effective responses, which can inform rational selection of antigen–adjuvant pairs. For example, integrative immunoprofiling combined with machine learning was applied to compare responses induced by two clinically relevant adjuvant systems (AS01B and AS02A) in a malaria subunit vaccine, revealing distinct cellular and antibody features that could be leveraged to tailor adjuvant choice for specific antigens and populations (47). Additionally, Bayesian optimization and ensemble learning guiding formulation have been used to assess antigen dose, adjuvant quantity, and timing when using mRNA lipid nanoparticle vaccines (48). Although these models have so far focused more on physical formulation parameters like particle size and encapsulation efficiency, they show how algorithmic optimization can reduce trial-and-error in designing vaccines with balanced potency and safety profiles.

### 4.4 Full replacement

In its most provocative sense, replacement implies the complete elimination of exogenous and traditional adjuvants. Recent experimental work provides proof-of-concept that such adjuvant-free vaccines are feasible under defined conditions. For instance, antigen–polymer conjugates designed to activate multiple innate immune pathways have elicited robust humoral and cellular immunity in the absence of added adjuvants. In SARS-CoV-2 and monkeypox subunit models, polymer–antigen constructs that engage both TLR and cGAS–STING pathways generated potent neutralizing antibodies and cytotoxic T-cell responses without external adjuvants, demonstrating that built-in innate stimulation can substitute for classical adjuvanticity (42). Preclinical studies of such protein nanoparticle vaccines have demonstrated durable antibody responses and robust T-cell activation driven by the multivalent display alone, suggesting that structural design features can substitute for chemical adjuvants.

## 5 Challenges

While these examples are promising, achieving true adjuvant replacement across diverse pathogens remains an aspirational goal. The immunogenicity afforded by built-in innate stimulation and optimized delivery varies between antigens and host contexts, and many of these technologies still rely on inherent immune activation rather than entirely novel mechanisms.

### 5.1 Biological complexity and data limitations

Currently, most AI or machine learning models are trained on relatively small and biased datasets (7–9, 27). Particularly, the data on innate immunity (PRR activation, inflammasome signaling, and cytokine networks) remain scattered and heterogeneous across clinical studies. Without standardized, high-dimensional datasets, model predictions risk overfitting or generalizing poorly to new pathogens and diverse human populations.

### 5.2 Safety and interpretability

As overstimulation of immunity can lead to severe side effects, classical adjuvants have faced decades of important regulatory scrutiny (1, 11). While AI-designed antigens with embedded adjuvant-like functions may outperform the conventional counterparts, such next-generation vaccine constructs could also introduce unforeseen safety concerns. Moreover, the black-box nature of many deep learning models limits mechanistic interpretability and may impede confidence among immunologists and regulatory authorities (9, 11).

### 5.3 Regulatory and ethical barriers

At present, no regulatory framework exists for the approval of an “AI-generated immune program” as a substitute for a chemical adjuvant. Key issues related to reproducibility, transparency, and liability remain unresolved. In addition, the concentration of AI infrastructure in high-income countries raises concerns about the potential exacerbation of global inequities in vaccine development and access (49–51).

## 6 A primary roadmap to gradually replace classical adjuvants with AI technologies

**Figure 2:**
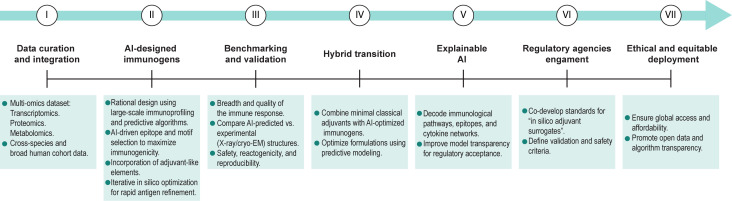
Conceptual roadmap toward the replacement of classical adjuvant with artificial intelligence-designed vaccines. This figure outlines a flexible roadmap for the development and validation of AI-designed immunogens with adjuvant-like functions. Key steps include (from left to right): (I) generation and curation of high-resolution, multi-omics immune datasets to train predictive AI models; (II) computational design of robust immunogens using large-scale immunoprofiling and predictive algorithms to identify epitopes, structural motifs, and molecular patterns that maximize immunogenicity while minimizing off-target responses; (III) benchmarking and experimental validation of AI-designed immunogens for humoral and cellular responses, antigen presentation, T-cell priming, and safety; (IV) hybrid transition strategies combining AI-guided immunogen design with minimal classical adjuvants as an intermediate phase; (V) implementation of explainable AI approaches to enhance mechanistic understanding and regulatory confidence; (VI) early engagement with regulatory agencies to establish validation frameworks for in silico adjuvant surrogates; and (VII) ethical and equitable deployment to ensure that resulting vaccines are globally accessible and reproducible. Collectively, these steps provide a conceptual and practical framework to guide the progressive transition toward AI-enabled vaccine innovation. Figure made with Illustrator.

To overcome the above challenges and move from a provocative concept to a practical implementation, I propose a basic and flexible roadmap that integrates advances in AI, immunology, and regulatory science (**Figure 2**):

### 6.1 Data generation and curation

Large-scale, standardized immunoprofiling initiatives (52) are needed to train AI systems with high-resolution immune data. Integration of transcriptomic, proteomic, metabolomic, and single-cell immune readouts across diverse human populations will allow models to predict immune stimulation with greater fidelity, providing the foundation for AI-driven vaccine design.

### 6.2 Computational design of robust immunogens

A central step in AI-driven vaccine development is the rational design of antigens using computational tools, as described in section 3. By leveraging large-scale immunoprofiling datasets and predictive algorithms, AI can identify epitopes, structural motifs, and molecular patterns that maximize immunogenicity while minimizing off-target or deleterious responses. Importantly, these highly optimized antigens provide the foundation for an eventual reduction in reliance on typical adjuvants, beginning with hybrid approaches and potentially culminating in formulations that no longer require exogenous adjuvants, which could shorten the timeline from conceptual design to functional validation.

### 6.3 Benchmarking and validation

Independent benchmarks, analogous to the Critical Assessment of Protein Structure Prediction (CASP) in structural biology, should be established for AI immunogenicity prediction (53, 54). Such efforts would set clear performance metrics and help distinguish robust approaches from overestimated claims. To experimentally assess the potential of AI-designed antigens to functionally replace classical adjuvants, targeted validation strategies should be implemented. For instance, AI-designed antigens with integrated adjuvant-like functions can be assessed for humoral and cellular immune responses along with antigen presentation, T-cell priming and safety profiles. In addition, state-of-the-art computational approaches could facilitate and strengthen such validations (e.g., AlphaFold 3 and virtual cells, as mentioned in section 3). Together, these complementary approaches would offer a rigorous basis to determine whether AI-generated antigens can recapitulate, or even have better immunostimulatory functions.

### 6.4 Hybrid transition strategies

In the near term, AI is more likely to minimize rather than completely replace adjuvants. For instance, computationally designed nanoparticles or antigens fused to PRR-ligand mimics may allow weaker, safer adjuvants to suffice.

### 6.5 Explainable AI

Future models must prioritize interpretability by identifying the motifs, pathways, or cytokine signatures that drive predictions. This will facilitate mechanistic validation *in vitro* and *in vivo*, bridging trust gaps with both scientists and regulators.

### 6.6 Regulatory engagement

Early dialogue with organizations such as the Food and Drug Administration (FDA) and European Medicines Agency (EMA) will be essential along with similar agencies in Latin America, Asia and Oceania. These global frameworks could accelerate approval avenues for “*in silico* adjuvant surrogates”, especially if these models can reduce animal testing.

### 6.7 Ethical and equitable deployment

To avoid exacerbating disparities, global consortia should ensure that AI infrastructure, training data, and resulting vaccines are accessible to low- and middle-income countries in an even manner with the industrialized counterparts.

## 7 Discussion

The idea that AI could replace classical adjuvants represents a paradigm shift in vaccinology, challenging assumptions about the permanent use of exogenous immune stimulants. Classical adjuvants have been indispensable due to their ability to orchestrate innate activation, antigen presentation, and adaptive immune polarization (11). Yet, these compounds are constrained by reactogenicity, species-specific responses, formulation complexity, and regulatory obstacles (6). AI-powered approaches offer a complementary and potentially transformative avenue by embedding adjuvant-like functions directly into vaccine design, thereby decoupling robust immune responses from chemical additives (7–9).

Current AI frameworks demonstrate adjuvant-mimicking capabilities across multiple areas: epitope prediction and structural modeling allow the design of antigens that naturally stimulate innate sensors, effectively reducing, at least partially, reliance on traditional adjuvants. Generative modeling, including transformer-based protein engineering, enables the incorporation of adjuvant-like motifs, such as PRR-ligand mimetics, directly into antigens. Data-driven simulations further predict the magnitude, polarization, and safety profile of immune responses, functioning as *in silico* surrogates for empirical adjuvant testing. Finally, computational screening of small molecules and *in silico* optimization of delivery platforms permits rational identification of immunostimulants and enhanced antigen presentation without conventional adjuvants (7–9, 27). Collectively, these approaches suggest that AI can replicate and even outperform multiple mechanistic roles of classical adjuvants.

Yet, several caveats temper enthusiasm: 1) Biological complexity remains a primary obstacle. Innate immune signaling is highly context-dependent, influenced by host genetics, microbiota composition, age, and gender, which makes accurate computational prediction challenging. 2) Data scarcity and heterogeneity further complicate model training, particularly for under-characterized PRR pathways and cytokine networks. 3) Additionally, interpretability is critical: deep learning approaches can limit mechanistic understanding and regulatory acceptance. 4) Safety concerns also remain salient as AI-guided designs could inadvertently trigger excessive inflammation, autoimmunity, or off-target effects.

To address these drawbacks, I propose a phased roadmap: 1) standardized, high-dimensional immunoprofiling across diverse human populations to improve model training; 2) benchmarked prediction frameworks akin to CASP for structural biology; 3) hybrid approaches combining AI-designed immunogens with minimal classical adjuvants as a transition strategy; 4) incorporation of explainable AI to facilitate mechanistic validation; and 5) early regulatory engagement and global equity considerations to ensure safe, reproducible, and broadly accessible implementation.

Importantly, this perspective does not encourage for the abandonment of classical adjuvants. Instead, it envisions a gradual, evidence-driven transition in which AI-enabled design increasingly complements and extends traditional approaches. The core functions of adjuvants—innate activation, antigen presentation, and immune modulation—can be replicated or even optimized computationally, potentially leading to vaccines that are safer, more efficacious, and faster to develop. Thus, while the full replacement of adjuvants remains speculative, current evidence highlights the plausibility of AI-driven “adjuvant surrogates” that can eventually reshape the conceptual and practical landscape of vaccinology.

In conclusion, AI is evolving from a supportive tool for epitope prediction to a potentially transformative technology capable of performing multiple adjuvant-like functions. Realizing the vision of AI-replaced adjuvants will require rigorous mechanistic validation, robust datasets, explainable models, and proactive regulatory and ethical frameworks. If successful, this paradigm could represent an era of rationally designed vaccines in which computationally embedded immunostimulation replaces empirical chemical adjuvants with remarkable human benefits.

## Data Availability

The original contributions presented in the study are included in the article/supplementary material. Further inquiries can be directed to the corresponding author.
